# Three Vital Subjects

**Published:** 1899-12

**Authors:** Lin. C. Shecut

**Affiliations:** Augusta, Ga.


ARTICLE V.

### THREE VITAL SUBJECTS.

LIN. C. SHECUT, D. D. S., AUGUSTA, GA.

Just lately, in the editorial columns of a dental journal the writer says: "For years we have held the opinion that a young man should not be encouraged by dentists to study dentistry, unless he has had a good preparatory literary education and has money enough to pay his way through college and "set himself up after graduation." This article brought to my mind the fact, that our journals have been teeming with writings relative to the requirements for entrance into the dental schools of this country; in our associations law upon law have been enacted, only to become "dead-letter" laws; the faculties of our colleges separately, and combined, have discussed it again and again only to return to their schools and entrust their sacred agreement to the fidelity and tender care of a grasping Dean, whose first question in his entrance examination is: "Have you the five dollars matriculation fee?" This being satisfactorily answered he puts his second and last. "When can you pay the tuition fee? In short, the profession in general has taken this as a topic ever ready for the literatures pen or the forensic fray. And what have we accomplished? Every year men are graduated from our colleges who are conspicuously deficient in the requirements of an elementary education. These men may do great good in their sphere, but they would have been capable of greater good to humanity, if it had been acquired that their attainments be greater. Still, on the other hand we find many with gifted faculties and cultivated mind; as fruitless as the barren fig-tree, when we consider their professional worth. And when thus we view the two, it would not take us long to decide which were the better filling his little "niche" in the world, and more deserving the help of the profession. One man making his one talent count, the other burying his five. Just so, we see how impossible it is for colleges to make men, and how easy for the spirit of the law to be perverted. Never-the-less, we agree with the editor on the question of preparatory literary education. But when he says "a young man should not start in dentistry unless he has money enough to pay his way through college and set himself up after graduation," we would beg to emphatically offer our objection. I will venture the statement, without fear of contradiction, that our best professional men to-day, are those who struggled, labored, and plodded between sessions, so that they might return the next. It is a notable fact, that our greatest minds have had their noblest thoughts burned into them by flickering candles and pine wood knots in attic rooms, and on cabin floors. Our illustrious men have been nutured in adversity's lap, and our genuises raised by the hand of poverty.

Let the young man plow the fields or strike at the forge, between sessions, if he feels that dentistry is his life-work, and this is the only way for him to get it. Don't discourage him, rather lend him a helping hand. Have confidence in him, and he will not disappoint you. So, one part of the article is good; the other I think goes to the extreme. And it strikingly shows us the strength and rapidity of a fast flowing current of human minds bent in one direction, and how easily they may run to an extreme that was not intended.

It should also serve as a reminder of our relationship and responsibility to the young men seeking our advice. How close is this relationship and how heavily does the responsibility rest on our shoulders. For after all, what are our professional relations but an intensified form of our relation man to man. In our days we hear so much and see so little of ethics. Our minds are muddled, we are un-uncertain and often ask "are there any ethical among us?" The natural laws that show us our duties to each other are simple and plain, and how far short of the true purposes does a man-made code fall. Many, often have the mistaken idea, that our laws are chiefly to regulate each other; to hold each other in obeyance and lose sight of the fact that they are made for the protection and benefit of humanity.

We should keep it clear in our minds that we are distinctly medical specialists, and that in our places, we have as great responsibility and owe as much to our fellows, as any other class. We must not allow the glittering gold and fine material necessary for our work, lead people to believe that our science has degenerated into an art, pursued mainly for pecuniary gain; but we must so act towards them and towards each other that the fact of our unselfish intent and noble purpose will be self-evident. In doing this, we should go beyond any arbitrary code, and must comprehend "that which is the most beautiful truth in morals, and that is: we are all dependently related." Where would you look for the man who is independent of his fellows? Is not greatness attained by serving? And are not those greatest that serve most? Our increased endowments, our extraordinary faculties, only lay upon us a greater weight of debt to others. We have no such thing as distinct or divided interests from our race. The Divine economy has it written—any evil that we practice or detriment that we work to our fellow man, will surely be revisited upon us, and poured upon our heads as coals of fire, even falling upon our children for generations to come. This of itself it seems would be sufficient to stop man in his stubbornness. But how much better to view the subject from the true and higher plane, where we do a thing, not through policy or fear of retribution, but from a love of the good and a desire for the right. Some one has beautifully put it that: "If we will but choose the broadest paths for the happiness of others, we unconsciously mark out our path through peace, prosperity and happiness." When God created man he manifestly put him here for his happiness. But man upset the plan, and so we have the world today, a mingled cup of sweet and bitter, and we add to this cup our share of the one or the other, just as we live for self or for our brother.

Often in casting about to find what we owe each to the other, we become tangled and confused with too much reasoning, and lose sight of the way that nature shows us; which is, in itself so simple, that we practice it unconsciously, and pervert it only as we will to do wrong. Look to our own bodies, and see how the organs have to work in sympathy with every other part, even to the minutest cell chamber, and just as the molecular disturbance in any one of these cells will cause confusion in some part, which, if not checked and righted, may lead to the destruction of the whole; just so is man the unit in the design of the universe, and the disturbance that he brings about will be felt in all those directions that his influence extends; and humanity will be lowered, and he receive his reward proportionately. Contemplate any of the wonderful workings of nature, even from the smallest of its making to those that seem as if they are separate words in themselves, and we find that each lives only by the help of its neighbor. They flourish only through God's universal law of dependent relation. From lowest life to highest, the silver cord runs, holding every part of creation in unison. Men realize this, why do they not live it? If we did, this old world would become as a grand symphony of songs, and life as the summer flight of a bird. Although it would be vain to look for this grand consummation here; still, it falls heavily upon every one, especially those striving for the higher plane of living, to look to this end. Doing so, life would be brighter and living sweeter. In every corner we would find concord, harmony and love. We would know, and live, natures ethics. Truly, "the world is what we make it." And in our sphere, be it far reaching or limited, we can approach this high ideal, if we will but strive.

